# The eCura system as a novel indicator for the necessity of salvage surgery after non-curative ESD for gastric cancer: A case-control study

**DOI:** 10.1371/journal.pone.0204039

**Published:** 2018-10-01

**Authors:** Hirotaka Niwa, Rie Ozawa, Yasunori Kurahashi, Tsutomu Kumamoto, Yasutaka Nakanishi, Koichi Okumura, Ikuo Matsuda, Yoshinori Ishida, Seiichi Hirota, Hisashi Shinohara

**Affiliations:** 1 Department of Surgery, Hyogo College of Medicine, Nishinomiya, Hyogo, Japan; 2 Department of Surgical Pathology, Hyogo College of Medicine, Nishinomiya, Hyogo, Japan; University of Florida, UNITED STATES

## Abstract

Endoscopic submucosal dissection (ESD) for early gastric cancer does not always lead to complete cancer resection. The aim of this study was to determine indicators for cancer residue (CR) status in cases of non-curative ESD. We analyzed 47 cases of non-curative ESD followed by salvage surgery and collected data regarding the rates of CR, which included both local CR and lymph node metastasis (LNM). To elucidate the risk factors for CR status, we compared the CR positive and the CR negative groups among surgical specimens according to the following variables obtained from ESD findings: tumor location, tumor size, depth of invasion, lympho-vascular invasion, histological margin, and histological diagnosis. The eCura system, which is an LNM risk scoring system, was also applied and scores were calculated in each case as follows: 3 points for lymphatic invasion and 1 point each for tumor size >30 mm, positive vertical margin, venous invasion, and submucosal invasion ≥500 μm. There were 9 (19%) CR positive cases, which included 6 cases of local CR and 4 cases of LNM; no cancer was detected in over 80% of the patients. The eCura scoring system was the only significant factor for CR status: the higher the eCura score, the greater the CR positivity (p = 0.0128). In particular, all patients in the low-risk group (score = 0–1 point) had no CR. Although no cancer recurrence was observed during a median follow-up of 4 years, 2 patients died of pneumonia. In conclusion, the eCura system might make it possible to select appropriate cases for salvage surgery.

## Introduction

Early gastric cancer (EGC) is defined as invasive gastric cancer that invades no deeper than the submucosa, irrespective of lymph node metastasis [1]. Endoscopic submucosal dissection (ESD), which allows en bloc resection of a whole layer of the mucosa with its dissection surface intact using connective-tissue coating [2], has been developed to become a minimally invasive and function-preserving treatment for EGC with negligible risk of lymph node metastasis (LNM) [3–6]. Because of these advantages, the indications for ESD have been expanding [6]. Eligibility for ESD consists of absolute indications and expanded indications, both categories of which are defined in the Japanese Gastric Cancer Treatment Guidelines 2014 (ver. 4) [7]. The curability of ESD is strictly estimated by histological assessment of the resected specimen [7]. When ESD for either an absolute or expanded indication is regarded as non-curative, salvage gastrectomy with concomitant lymphadenectomy is usually recommended to avert the risks of regional LNM [7]. However, the rate of LNM is not high and is reported to be 5–10% in patients who underwent salvage surgery for non-curative ESD [8]. In patients with gastric cancer, especially those with early cancer, background factors such as age, nutrition status, and comorbidities often influence the prognosis. Surgery can have detrimental effects on the patients’ quality of life [9], which surgeons sometimes underestimate. Clarifying the risk factors affecting CR status after non-curative ESD would be valuable to avoid unnecessary treatment such as ineffectual surgery.

Hatta *et al*. have recently established the “eCura” scoring system for predicting cancer-specific survival of patients with non-curative ESD [8]. This system was originally developed for prediction of LNM and consists of five factors, which are lymphatic invasion, tumor size, positive vertical margin, venous invasion, and submucosal invasion [8]. According to this scoring system, patients are categorized into three LNM risk groups: low (0–1 point: 2.5% risk), intermediate (2–4 points: 6.7%), and high (5–7 points: 22.7%) [8]. The relationship between the eCura score and cancer-related outcome for patients with non-curative ESD has previously been discussed [10]. Accordingly, benefits of salvage surgery can be expected in the high-risk group and follow-up alone is sufficient in the low-risk group [10]. In contrast, the treatment strategy in the intermediate group is indeterminate [10].

In this study, we investigated the rates of local CR and LNM in patients with gastric cancer who underwent salvage gastrectomy for non-curative ESD and applied the eCura scoring system to our data and explored whether it could serve as an indicator in the selection of appropriate patients for salvage surgery.

## Materials and methods

### Study design and ethics statement

This was a retrospective case-control study performed in a single institute. The study protocol was approved by the Institutional Medical Ethics Committee (approval number 2753). The Committee waived the requirement for informed consent for this study.

### Patients

Among patients undergoing ESD, 47 patients who underwent salvage surgery for non-curative ESD between 2009 and 2015 were enrolled.

### ESD techniques

The ESD procedure as practiced in our institute was reported previously [11]. ESD was performed using an insulation-tipped diathermic (IT-2) knife (KD-610L; Olympus Medical Systems, Tokyo, Japan) and ball-tipped FlushKnife (FlushKnife-BT) (DK2618JB/DK2618JN; Fujifilm Medical Co., Ltd., Tokyo, Japan). We marked the normal mucosa 5 mm outside the tumor edge with a needle knife (KD-1L-1; Olympus). Saline with adrenaline (1:10 000 solution in saline) was injected into the submucosa. The mucosa 5 mm outside the mark was cut circumferentially using a VIO electrosurgical generator (Erbe, Tübingen, Germany).

### Salvage gastrectomy

Gastrectomy with concomitant lymph node dissection is recommended by the guidelines, in principle, after non-curative ESD [7]. Postoperative complications were assessed according to the Clavien-Dindo (CD) classification [12] and complications classified as II or worse were listed in this present study.

### Pathological evaluation

All specimens were completely dissected into 2- to 3-mm thick slices. Then, 3-μm sections from each slice were examined with hematoxylin and eosin (HE) or Elastica van Gieson staining [13]. Immunohistochemistry for desmin and D2-40 was performed to visualize muscularis mucosae and lymphatic vessels, respectively [13].

Histological subtype, depth of tumor invasion, and lymph node metastasis were evaluated according to the Japanese Classification of Gastric Carcinoma: 3^rd^ Edition [1].

Histological subtypes are largely classified into two groups, differentiated and undifferentiated types [7]. The differentiated type includes papillary adenocarcinoma (designated “pap”) and tubular adenocarcinoma (designated “tub”). The “tub” category is further subdivided into “tub1” (well differentiated) and “tub2” (moderately differentiated) [7]. The undifferentiated group includes signet-ring cell carcinoma (designated “sig”) and poorly differentiated adenocarcinoma (designated “por”). The “por” category is further subdivided into “por1” (medullary) and “por2” (diffuse or scirrhous) [7]. If mucinous adenocarcinoma component was found in the submucosal layer, ESD was defined as non-curative [7].

The curability of ESD was assessed according to the Japanese Gastric Cancer Guidelines 2014 (ver. 4) [7]. Cases with the possibility of positive resection margin were included in the positive margin group.

### Data collection

Pathological and clinical outcomes of the 47 enrolled patients were analyzed retrospectively. The following variables were assessed: age at time of surgery, sex, tumor location, pathological invasion depth diagnosed by findings obtained from ESD and surgical specimens, greatest tumor diameter, main histological type in the ESD specimens, lymphovascular invasion, resection margins (horizontal margin and vertical margin), surgical procedures, postoperative complications, and outcomes.

We compared the CR positive group with the CR negative group according to the following variables obtained from ESD findings: tumor location, tumor size, depth of invasion, lympho-vascular invasion, histological resection margins and histological diagnosis, and eCura scores.

### eCura system

eCura scores were calculated in each case as follows: 3 points for lymphatic invasion and 1 point each for tumor size >30 mm, positive vertical margin, venous invasion, and deep submucosal invasion ≥500 μm (SM2) [8]. In this scoring system, patients are categorized into three LNM risk groups based on the scores, namely, low risk (0–1 points: 2.5% LNM risk), intermediate risk (2–4 points: 6.7%), and high risk (5–7 points: 22.7%) [8].

### Statistical analysis

Fisher’s exact probability test was performed for categorical variables between the two groups. For all tests, all *P* values were two-sided, and *P* < 0.05 was considered statistically significant.

## Results

### Baseline characteristics and clinicopathological data

Table 1 shows the patients’ basic characteristics, tumor location, tumor size, tumor invasion depth, histological type of tumor in ESD specimens, indications for salvage surgery, surgical procedures, postoperative complications, and outcomes. More than 39 (80%) tumors were located in the middle and lower parts of the stomach. Depth of tumor invasion was classified as mucosal invasion (M) in 5 cases (11%), superficial submucosal invasion (SM1) in 7 (15%), deeper submucosal invasion (SM2) in 32 (68%), muscularis propria invasion (MP) in 2 (4%), and subserosal invasion (SS) in 1 (2%). Around two-thirds (34) of the cases were included in the differentiated type on histology. The most common indication for salvage gastrectomy was SM2 invasion (35) followed by lymphatic invasion (21). Partial gastrectomy was selected in 44 cases (over 90%) and total gastrectomy was performed in only 3 cases. Postoperative complications classified as II or worse on the CD scale were observed in 7 cases (3 complications of anastomosis, 2 small bowel obstruction, and 2 pancreatic fistula). The cancer-related prognosis was favorable and no cancer recurrence was observed during a median follow-up period of 4 years (range 2–8 years). Two patients died of benign disease (pneumonia).

**Table 1:** Baseline characteristics and clinicopathological information of the 47 patients who underwent salvage surgery for non-curative ESD.

Age	Range, median (years)	36–83,71
Sex	Male/Female	34/13
Tumor location	Upper/Middle/Lower	8/22/17
Tumor size	Range, median (mm)	7–49, 17
Invasion depth	M/SM1/SM2/MP/SS	5/7/32/2/1
Dominant histology	Differentiated (pap, tub1, tub2)	34
	Undifferentiated (por, sig)	12
	Unclassified (muc)	1
Indications for salvage surgery	SM2 invasion	35
	Lymphatic invasion positive	21
	Vertical margin positive	20
	Vascular invasion positive	16
	Horizontal margin positive	4
Surgical procedure	Total gastrectomy/others	3/44
Postoperative complication	Anastomotic trouble	3
	Small bowel obstruction	2
	Pancreatic fistula	2
Long-term outcome in median	Cancer recurrence	0
follow-up of 4 years	Death (cause)	2 (pneumonia)

### Cancer residue in the surgical specimens

Table 2 shows the CR status of specimens from salvage surgery. CR was detected in 9 cases (19.1%). These consisted of 6 cases of local CR and 4 of LNM. One case had both local CR and LNM.

**Table 2:** CR status in the surgical specimens.

Cancer residue status	Positive (n = 9)	Negative (n = 38)
Only local CR	5	N/A
Only LNM	3	N/A
Both local CR and LNM	1	N/A

N/A, not applicable

Table 3 shows the results of the comparison between the CR positive and the CR negative groups. Variables such as tumor location, tumor size, depth of invasion, lymphovascular invasion, histological margins, and histological type were not significant. Only the eCura scoring system was significantly different between the two groups (*P* = 0.0128).

**Table 3:** Comparison of clinicopathological findings between the CR positive and CR negative groups.

		CR positive (n = 9)	CR negative (n = 38)	P value
Tumor location	Upper	2	6	0.639
	Middle+Lower	7	32	
Tumor size, mm*	≤30 mm	5	29	0.190
	30 mm<	4	7	
Depth of invasion	M, SM1	0	12	0.087
	SM2 or deeper	9	26	
Lymphatic invasion	Negative	3	23	0.263
	Positive	6	15	
Vascular invasion	Negative	5	26	0.466
	Positive	4	12	
Vertical margin	Negative	4	24	0.453
	Positive	5	14	
Horizontal margin	Negative	7	36	0.161
	Positive	2	2	
Dominant histology	Differentiated	6	28	0.692
	Undifferentiated+muc	3	10	
eCura scores*	Low risk	0	12	
	Intermediate risk	4	19	0.0128**
	High risk	5	5	

Differences were analyzed by Fisher’s exact test.

*Two cases were excluded due to missing data regarding tumor size.

* * Statistically significant

**Fig 1:**
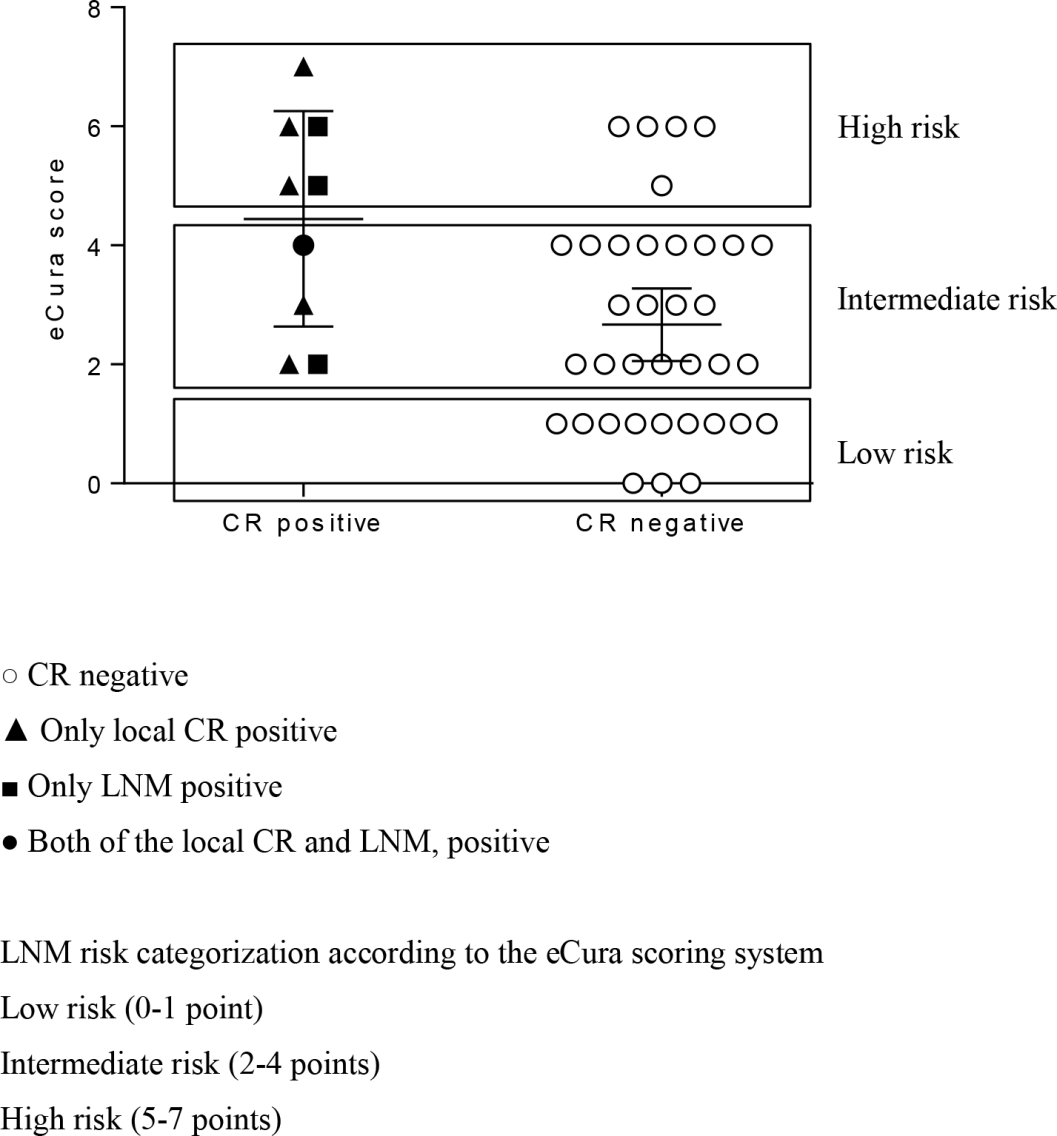
Relationship between eCura scores and CR status. Each symbol represents 1 patient. The long and short vertical bars represent the mean and mean ± standard deviation, respectively. According to the eCura risk scoring system, CR positivity rates were 0% (0/12), 17% (4/23), and 50% (5/10) in the low-risk group, intermediate-risk group, and high-risk group, respectively.

Fig 1 shows the relationship between eCura scores and CR status. CR positivity rates were 0% (0/12), 17% (4/23), and 50% (5/10) in the low-, intermediate-, and high-risk groups, respectively.

**Fig 2:**
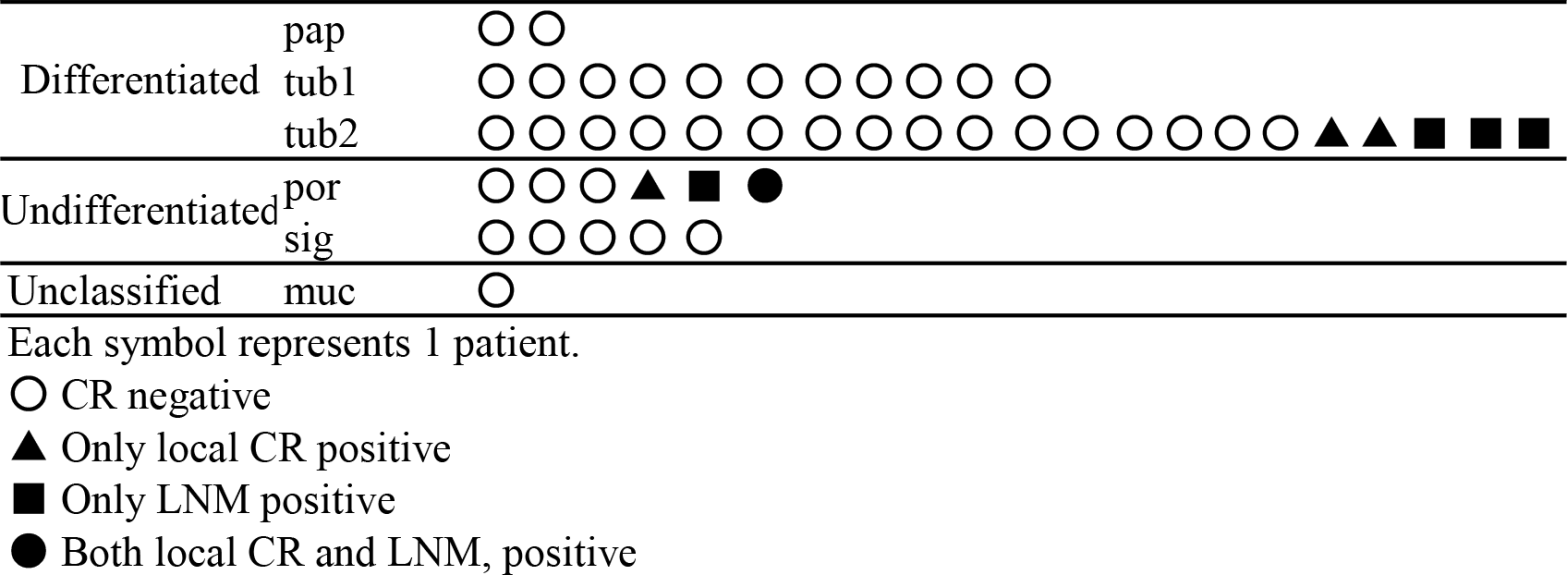
Relationship between histological type and CR status in the 46 cases with en bloc ESD. Fig 2 shows the relationship between the main histological types and CR status in the 46 cases with en bloc ESD. One case was excluded due to possible inaccuracy of histological diagnosis because of piecemeal ESD. Positive CR is recognized in 8 patients, which included 3 cases of LNM, 4 cases of local CR, and 1 case of both local CR and LNM. Note that CR was positive in only the cases of moderately differentiated type (tub2) in the differentiated group.

Fig 2 shows the relationship between the main histological types and CR status in the 46 cases with en bloc ESD. One case was excluded due to possible inaccuracy of histological diagnosis because of piecemeal ESD. Eight cases were CR positive and they included the 3 LNMs and the 4 local CRs. One case had both local CR and LNM. Note that, in the differentiated group, CR was positive in only cases of moderately differentiated type (tub2). Thus, no CR was detected in patients with papillary and well-differentiated adenocarcinoma type.

## Discussion

Recently, ESD has become a vital treatment option for EGC with negligible risk of LNM. The prognosis following ESD is favorable, and the 5-year disease-specific survival rate was reported to be nearly 100% in patients who underwent curative resection [14]. However, according to the Japanese nationwide registry survey conducted in 2013, non-curative endoscopic resection accounted for 29.3% of all ESD procedures [14]. Regarding treatment for non-curative ESD, options include salvage surgery and additional endoscopic procedures [7]. Endoscopic treatment such as re-ESD or endoscopic coagulation may be possible for patients with positive horizontal margins of the differentiated type without lymphatic invasion [15]. The aim of salvage surgery is to avert possible LNM and its benefit has been discussed previously [5, 16]. However, some of recent reports suggested that follow-up without surgery might be acceptable in certain conditions, judging from the low rate of LNM in EGC, even when the pathological results of ESD did not meet the curative criteria [17, 18]. In fact, according to the report of the nationwide registry in Japan, no additional treatment was selected in more than 60% of non-curative ESD cases [14].

The current study revealed that the rate of LNM in non-curative ESD was 8.5%, which is consistent with previous studies at 5–10% [8]. In addition to the low possibility of LNM, 5-year disease-specific survival was reported to be 98.7% even in patients who underwent non-curative ESD [14]. Conversely, surgery-related complications classified as II or worse on the CD scale occurred in 7 patients (14.9%) in this study. Although no cancer recurrence was observed during a median follow-up of 4 years, 2 patients (4%) died of pneumonia. Therefore, taking into consideration the low LNM rate, risk of surgery, and the promising good prognosis, salvage surgery for all patients could be regarded as excessive, and so novel indicators for CR status are required.

We compared nine clinicopathological factors between the CR positive group and the CR negative group to determine indicators for CR status. From our results, the eCura scoring system was the only significant factor for CR positivity. This system was originally established as an LNM risk scoring system and was reported to be associated with cancer prognosis after ESD [8, 10]. Hatta et al. reported the usefulness of this system for determination of indications for salvage surgery after non-curative ESD [10]. They discussed the benefits of salvage surgery in comparison with no additional treatment for non-curative ESD in accordance with risk categories [10]. In the high-risk category, since cancer-specific survival was higher in the salvage surgery group than the follow-up group, the benefits of surgery seemed to be positive [10]. Among patients treated at our hospital during the study period who declined salvage surgery for non-curative ESD, there were 3 patients in the high-risk category, 1 of whom had metastasis to the paragastric lymph node (data not shown).

However, in the low-risk category, no significant difference in survival was seen between the salvage surgery group and the follow-up group [10]. Therefore, follow-up without surgery might be acceptable in the low-risk group [10]. In contrast, the benefits of salvage surgery were ambiguous and treatment strategy was indeterminate in the intermediate-risk category [10]. In the present study, CR positivity status appeared to be associated with eCura scores. Notably, all the 12 cases with low risk scores (0–1 points) had no CR (Fig 1). This result was consistent with Hatta’s suggestion that patients with low risk need not undergo salvage surgery.

Regarding tumor depth, SM2 invasion turned out to be insignificant with respect to CR status, although all CR positive cases were in the SM2 invasion group. According to a previous study that investigated outcomes of ESD for submucosal invasive gastric cancer, no significant difference between SM1 invasive cancer and SM2 was found with regard to rates of LNM and cancer recurrence [19]. Therefore, careful follow-up might be acceptable for patients with EGC whose non-curative factor is limited solely to SM2 invasion (eCura score = 1), especially when factors such as age, and comorbidities are taken into consideration. The rates of LNM and cancer recurrence for these patients are reported to be 2.5% and 0.7%, respectively [19].

In judging eligibility for ESD, the histological type of EGC is usually divided into two groups comprising the differentiated and undifferentiated types [7]. The tub2 type is usually included in the former along with pap and tub1 [7]. However, tub2 is often associated with mixed histology, which was reported to be a risk factor for LNM in EGC [20]. Moreover, a previous report hypothesized that tub2 might have the possibility of dedifferentiation during the growth process [21]. Therefore, we supposed that this usual histological division might not be appropriate when considering additional treatment for patients with non-curative ESD. As illustrated in Fig 2, all the CR positive cases in the differentiated type were included in the tub2 type group. We speculated that tub2 might have different features from tub1 and pap. Therefore, caution should be exercised in treatment decisions for patients with a diagnosis of tub2.

There were several limitations in this study. Our study had a retrospective design in a single institute and evaluated only patients who underwent salvage surgery. Also, the sample size was small. Thus, further large-scale analysis should be considered.

## Conclusions

Over 80% of patients undergoing salvage surgery for non-curative ESD did not have CR. The eCura system appeared helpful in determining whether to add salvage surgery.
